# Impact of Oligoether Side-Chain Length on the Thermoelectric Properties of a Polar Polythiophene

**DOI:** 10.1021/acsaelm.3c00936

**Published:** 2023-09-15

**Authors:** Mariavittoria Craighero, Jiali Guo, Sepideh Zokaei, Sophie Griggs, Junfu Tian, Jesika Asatryan, Joost Kimpel, Renee Kroon, Kai Xu, Juan Sebastian Reparaz, Jaime Martín, Iain McCulloch, Mariano Campoy-Quiles, Christian Müller

**Affiliations:** † Department of Chemistry and Chemical Engineering, Chalmers University of Technology, Goteborg 41296, Sweden; ‡ Materials Science Institute of Barcelona, ICMAB-CSIC, Campus UAB, 08193 Bellaterra, Spain; § Department of Chemistry, University of Oxford, Chemistry Research Laboratory, 12 Mansfield Road, Oxford OX1 3TA, United Kingdom; ∥ Universidade da Coruña, Campus Industrial de Ferrol, CITENI, Esteiro, 15403 Ferrol, Spain; ⊥ Laboratory of Organic Electronics, Linköping University, 60174 Norrköping, Sweden; # POLYMAT, Paseo Manuel de Lardizabal 3, 20018 Donostia-San Sebastián, Spain

**Keywords:** conjugated polymer, side-chain length, organic thermoelectrics, chemical doping, electrical conductivity

## Abstract

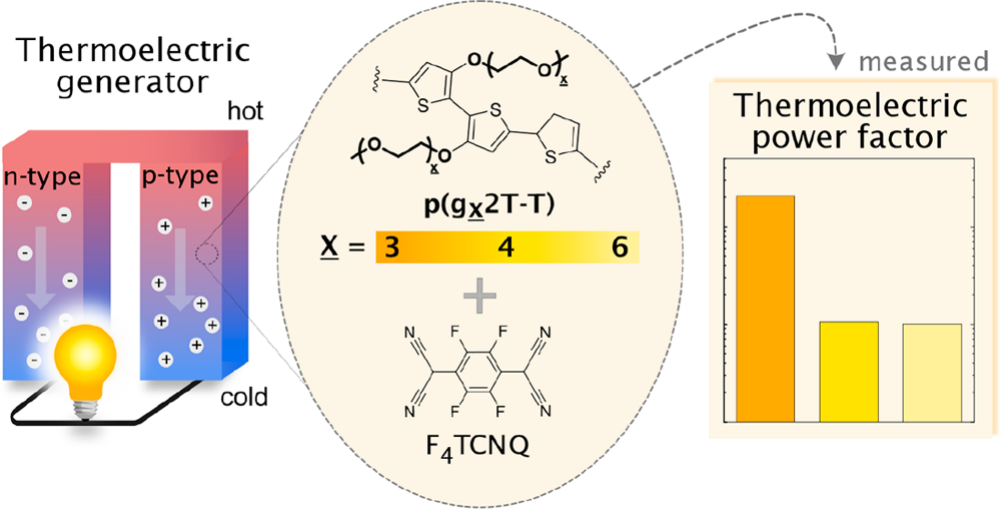

Conjugated polymers with oligoether side chains make up a promising class of thermoelectric materials. In this work, the impact of the side-chain length on the thermoelectric and mechanical properties of polythiophenes is investigated. Polymers with tri-, tetra-, or hexaethylene glycol side chains are compared, and the shortest length is found to result in thin films with the highest degree of order upon doping with the p-dopant 2,3,5,6-tetrafluoro-7,7,8,8-tetracyanoquinodimethane (F_4_TCNQ). As a result, a stiff material with an electrical conductivity of up to 830 ± 15 S cm^–1^ is obtained, resulting in a thermoelectric power factor of about 21 μW m^–1^ K^–2^ in the case of as-cast films. Aging at ambient conditions results in an initial decrease in thermoelectric properties but then yields a highly stable performance for at least 3 months, with values of about 200 S cm^–1^ and 5 μW m^–1^ K^–2^. Evidently, identification of the optimal side-chain length is an important criterion for the design of conjugated polymers for organic thermoelectrics.

## Introduction

Organic thermoelectric materials have recently gained considerable attention, because they can be used to construct flexible and low-cost thermoelectric devices that can directly convert heat to electricity. Thermoelectric devices could operate as autonomous power sources that run the myriad of distributed microelectronic devices, which are to make up the Internet of Things (IoT).^1−3^ Suitable thermoelectric materials should display a high figure of merit *ZT* = α^2^σ*T*/κ, where α is the Seebeck coefficient, σ and κ are the electrical and thermal conductivity, and *T* is the absolute temperature. Conjugated polymers are appealing candidates for thermoelectrics, as they combine ease of processing and a low thermal conductivity with advantageous mechanical properties and low weight. These characteristics make them suitable for the fabrication of cost-effective wearable energy harvesting devices.^2,4^

Significant research efforts are being dedicated to improving the thermoelectric performance of organic semiconductors. There are two main approaches for increasing the figure of merit that focus on reducing κ or enhancing the power factor α^2^σ. Typically, the thermoelectric parameters cannot be optimized independently. Materials should be selected that combine a low thermal conductivity with a high charge-carrier mobility. The ideal material would embody the “phonon-glass electron-crystal” concept.^5^ Further, the chosen processing technique and the resulting nanostructure strongly influence the thermoelectric parameters. For example, the use of porous structures can reduce κ but also negatively affect σ,^6,7^ while uniaxial orientation can significantly increase σ as well as κ.^8^ It is possible to reach a high electrical conductivity through chemical doping, and the selection of the right dopant and doping process not only allows to maximize the electrical conductivity for a given polymer:dopant pair but also the power factor.^9,10^ Intriguingly, doping can either increase or decrease κ^11−13^ by enhancing the electronic contribution to heat transport or by inducing a solid solution scattering effect, respectively. This dual effect of chemical doping provides a potential means to decouple the thermoelectric parameters and optimize the figure of merit.

The most common architecture of solution-processable polymer semiconductors comprises a conjugated backbone that is decorated with solubilizing side chains.^14,15^ Alkyl side chains are selected to achieve solubility in organic apolar solvents, while oligoether side chains lead to polymers that can be processed from more polar solvents. Moreover, polar side chains improve the compatibility between the dopant and the semiconductor host. For example, the polythiophene p(g_4_2T-T) with tetraethylene glycol side chains displays good compatibility with, e.g., the strong oxidant 2,3,5,6-tetrafluoro-7,7,8,8-tetracyanoquinodimethane (F_4_TCNQ) as well as acid dopants, leading to an electrical conductivity as high as 100 S cm^–1^.^16,17^ A similar compatibilizing effect has been observed for n-type organic semiconductors. Both fullerenes and polymers with oligoether pendant groups display enhanced miscibility with dopants such as 4-(2,3-dihydro-1,3-dimethyl-1*H*-benzimidazol-2-yl)-*N*,*N*-dimethylbenzenamine (N-DMBI).^18,19^

**Figure 1:**
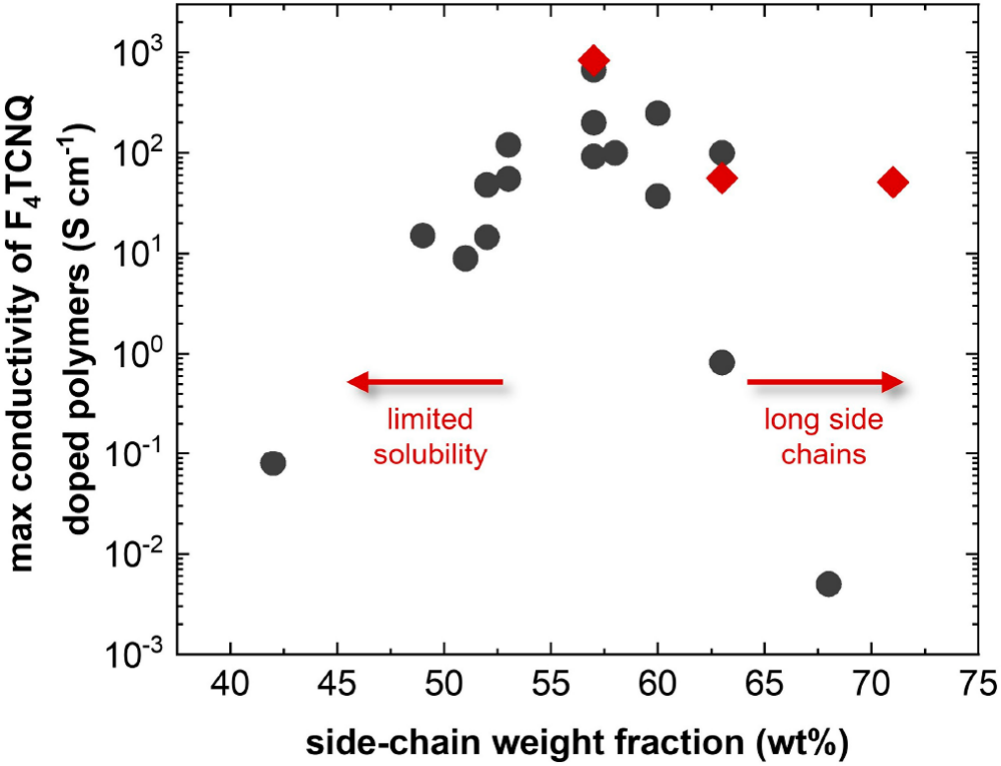
Maximum electrical conductivity values reported for polythiophenes and thienothiophene-based copolymers doped with F_4_TCNQ^9,16,20−22,25−33^ (black circles), and values obtained in this study (red diamonds).

For many types of polymers, there is an optimal side-chain length: too short side chains hamper processability, while too long side chains dilute the volume fraction occupied by charge-conducting backbones, often leading to a nanostructure that is not favorable for charge transport (Figure 1). For example, the presence of long side chains leads to a low electrical conductivity, since the volume of charge-conducting material is unduly diluted but also because the resulting polymers tend to be less ordered. Poly(3-dodecylthiophene) (P3DDT) with 68 wt % of side chains with respect to the molecular weight of the repeat unit shows an electrical conductivity of not more than 0.005 S cm^–1^,^20^ while poly(3-hexylthiophene) (P3HT) with 52 wt % of side chains has an electrical conductivity of up to 48 S cm^–1^ when doped with F_4_TCNQ.^21^ On the other hand, a polymer with short side chains can suffer from poor processability, and therefore, it may be difficult to obtain a nanostructure that is favorable for charge transport. Poly(3-butylthiophene) (P3BT) has a side-chain weight fraction equal to 42 wt % and displays an electrical conductivity of not more than 0.08 S cm^–1^ when doped with F_4_TCNQ.^22^ Further, it can be anticipated that the side-chain fraction influences the mechanical properties of the polymers,^23,24^ which must be carefully selected if free-standing and/or mechanically robust devices are envisaged.

**Figure 2:**
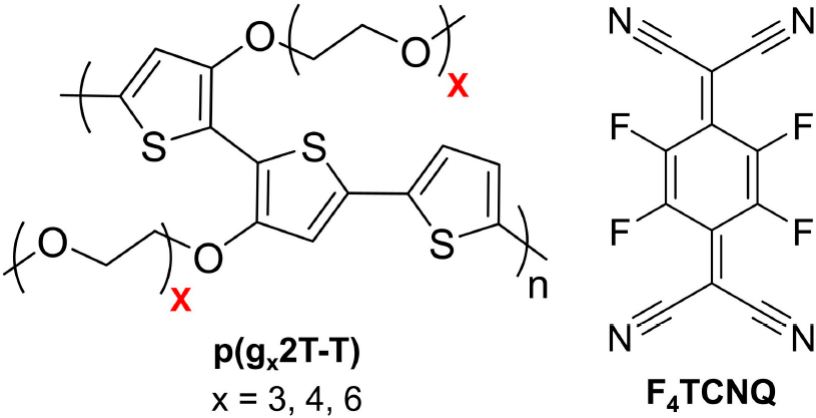
Molecular structures of p(g_*x*_2T-T) and F_4_TCNQ.

Here, we investigate how the side-chain length impacts the thermoelectric performance and mechanical properties of polythiophenes with oligoether side chains. We focus on a family of three polymers based on the repeat unit g_*x*_2T-T (see Figure 2),^14,16^ herein referred to as p(g_*x*_2T-T) with *x* = 3, 4, or 6, corresponding to a side-chain fraction of 57, 63, and 71 wt %. Previous studies have shown that shorter side chains, i.e., *x* = 2, result in a barely soluble and thus largely intractable material,^14^ and therefore, polymers with *x* < 3 were excluded. A significant impact of the side-chain length is observed, with p(g_3_2T-T) displaying the highest electrical conductivity and thus thermoelectric performance upon doping with the model dopant F_4_TCNQ (Figure 1).

## Results and Discussion

In a first set of experiments, we investigated the extent of oxidation of thin films of p(g_*x*_2T-T) polymers coprocessed with 20 mol % of F_4_TCNQ per thiophene ring using UV–vis and FTIR spectroscopy. This dopant concentration was chosen because previous studies have shown strong oxidation of p(g_4_2T-T) while maintaining a favorable nanostructure for charge transport.^20^ Doping of p(g_*x*_2T-T) type polymers with side chains other than tetraethylene glycol has not yet been investigated. Thus, we carried out a series of experiments involving coprocessing of p(g_3_2T-T) with F_4_TCNQ, which confirmed that 10–30 mol % of the dopant results in a consistently high electrical conductivity of more than 200 S cm^–1^ (Figures S1 and S2).

**Figure 3:**
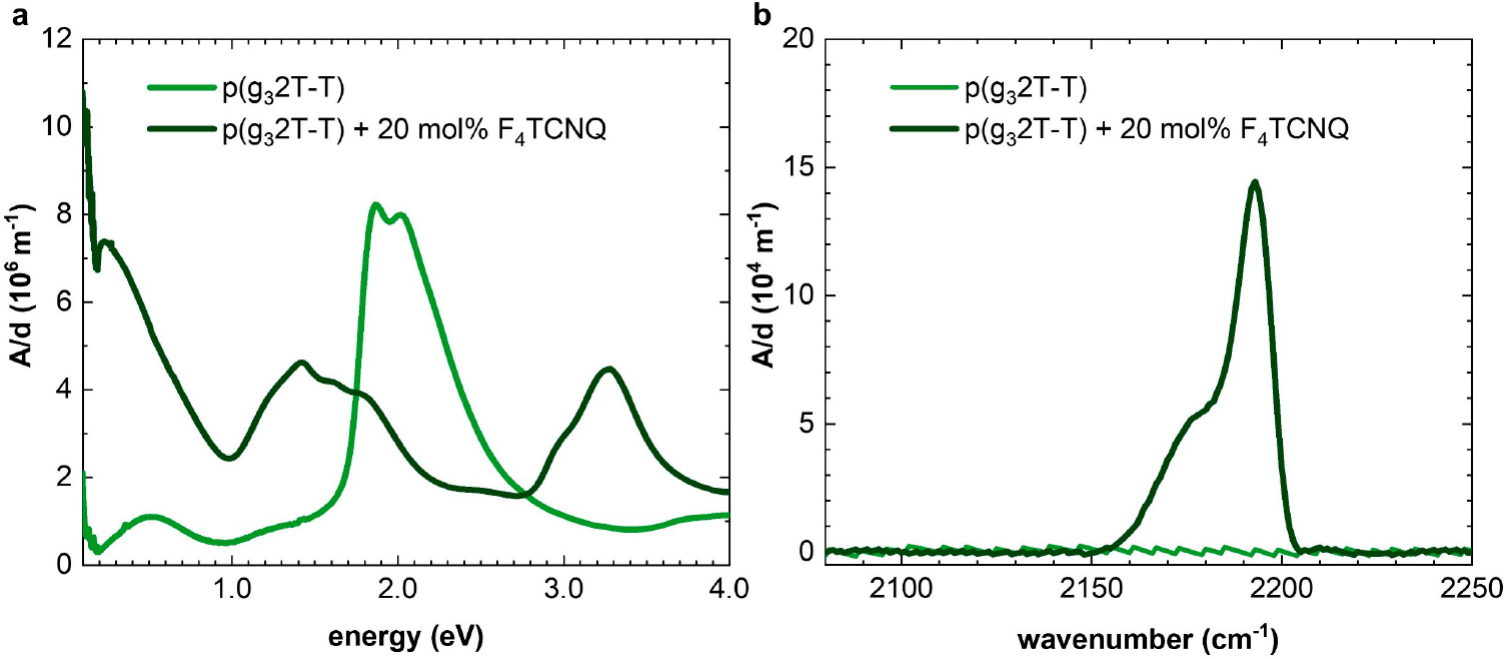
UV–vis (a) and transmission FTIR (b) absorbance spectra, with the absorbance *A* normalized by the film thickness *d*, of p(g_3_2T-T) before (light green) and after coprocessing with 20 mol % F_4_TCNQ (dark green).

The UV–vis spectra of all three neat polymers feature absorption peaks (or shoulders) at 1.9 and 2 eV, with the former being most pronounced in the case of p(g_3_2T-T) (Figures 3 and S3). The absorption peak at 1.9 eV likely indicates, in analogy to, e.g., P3HT, ordering of the polymer. Interestingly, p(g_6_2T-T) exhibits the weakest absorption intensity, which can be rationalized with its larger side-chain fraction. Additional absorption peaks, located in the near-infrared (NIR) region at 0.5 eV and around 1.3 eV in case of p(g_3_2T-T) (Figure 3), are observed, which arise due to adventitious oxidation of the backbone by atmospheric oxygen^34^ resulting in a weakly doped polymer.^35^

The absorption spectra of the three polymers coprocessed with 20 mol % of F_4_TCNQ do not show any significant absorption arising from the neat material (Figures 3 and S3), indicating strong oxidation of the backbones. The backbone oxidation is further confirmed by the appearance of distinct polaronic absorption peaks in the NIR region of the spectra as well as two additional peaks around 1.5 eV that are characteristic for F_4_TCNQ anions. The presence of ionized dopant molecules is also supported by the appearance of absorption peaks at 2190 cm^–1^ in the infrared region, which can be assigned to the cyano stretch vibration of F_4_TCNQ^–^.^32^ Moreover, the spectra of all p(g_*x*_2T-T) polymers coprocessed with 20 mol % of F_4_TCNQ films display an absorption peak at 3.4 eV, indicating the presence of neutral dopant species. The absence of a peak at 3.7 eV (Figure 3a) and the absence of cyano stretch vibration peaks characteristic for F_4_TCNQ^2–^ (Figure 3b) suggest that no significant amount of dianions is present, as expected for high F_4_TCNQ concentrations.^32^

By comparing the absorption peaks around 3.4 eV in the UV–vis spectra of doped films with absorption spectra previously reported for neutral F_4_TCNQ and its anion in dry acetonitrile solution, we assessed the ratio of ionized and neutral dopant molecules *f* that are present in the films (Figure S4),^32^ which allowed us to estimate the ionization efficiency η_*ion*_ = *f*/(1 + *f*) and number of charge carriers, i.e., polarons, *N*_*p*_^*UVvis*^ per unit volume (see Supporting Information), assuming that each F_4_TCNQ anion generates one polaron on the polymer backbone. Among the three polymers coprocessed with 20 mol % F_4_TCNQ, p(g_3_2T-T) shows the highest values, i.e., η_*ion*_^*UVvis*^ = 42 ± 14% and *N*_*p*_^*UVvis*^ = 2.8 ± 0.8 · 10^26^ m^–3^ (Table 1). We also used FTIR to estimate the number of polarons (*N*_*p*_^*FTIR*^) of the samples coprocessed with 20 mol % dopant by comparing the relative intensities of the cyano stretch vibration peaks at 2190 cm^–1^ with the extinction coefficient previously reported for F_4_TCNQ-doped p(g_4_2T-T) with an ionization efficiency of 100% (Table S1).^20,32^ Assuming that the extinction coefficient scales linearly with the concentration of ionized dopant in the sample and that each anion generates one polaron, a value of η_*ion*_^*FTIR*^ = 31 ± 9% is obtained for p(g_3_2T-T), in good agreement with η_*ion*_^*UVvis*^. Instead, for p(g_4_2T-T) and p(g_6_2T-T), we estimated values that are approximately twice those obtained from the analysis of UV–vis spectra (Table S1).

**Table 1:** Electrical Properties of Thin Films Coprocessed with 20 mol % F_4_TCNQ per Thiophene Ring^a^

polymer	η_*ion*_^*UVvis*^ (%)	*N*_*p*_^*UVvis*^ (10^26^ m^–3^)	σ (S cm^–1^)	μ (cm^2^ V ^–1^ s^–1^)	α (μV K^–1^)	α^2^ σ (μW m^–1^ K^–2^)
p(g_3_2T-T)	42 ± 14	2.8 ± 0.8	830 ± 15	18.7 ± 5.6	15.8 ± 2.0	20.7 ± 3.8
p(g_4_2T-T)	36 ± 12	2.1 ± 0.6	56 ± 3	1.7 ± 0.5	13.8 ± 0.5	1.1 ± 0.1
p(g_6_2T-T)	28 ± 9	1.4 ± 0.4	51 ± 4	2.3 ± 0.7	14.1 ± 0.7	1.0 ± 0.1

a Polymer, ionization efficiency η_*ion*_^*UVvis*^ and number of polarons per unit volume *N*_*p*_^*UVvis*^ from analysis of UV-vis spectra (estimated error of 30% based on uncertainty in thickness measurement and the analysis of UV-vis spectra), electrical conductivity σ (error represents the standard deviation of five measurements on the same sample), charge-carrier mobility μ, Seebeck coefficient α (error represents the standard deviation of five measurements on the same sample), and power factor α^2^σ. All spectroscopy and electrical conductivity measurements were carried out using the same set of samples (see Figure S2 for repeat measurements of σ and α).

**Figure 4:**
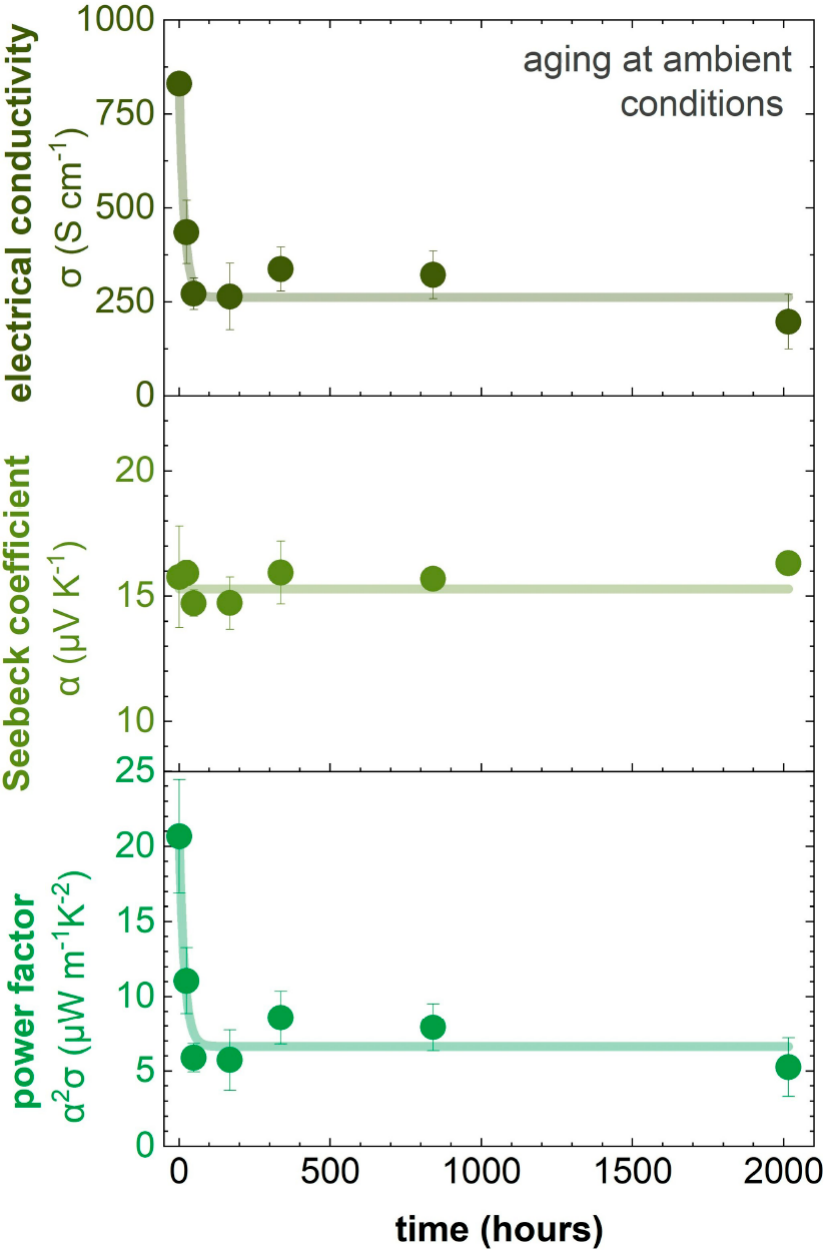
Electrical conductivity σ, Seebeck coefficient α, and power factor α^2^σ versus aging time of p(g_3_2T-T) coprocessed with 20 mol % of F_4_TCNQ at ambient conditions; error bars represent the standard deviation of five measurements on the same sample.

We measured the electrical conductivity of films of p(g_*x*_2T-T) coprocessed with 20 mol % F_4_TCNQ and observed a significant influence of the side-chain length. P(g_3_2T-T) displays the highest electrical conductivity among the three analyzed polymers, reaching a value of σ = 830 ± 15 S cm^–1^ (Figure 4, Table 1). Instead, p(g_4_2T-T) and p(g_6_2T-T) show comparable values of σ = 56 ± 3 and 51 ± 4 S cm^–1^, respectively (Figure S5, Table 1). Using the obtained values for σ and *N*_*p*_^*UVvis*^, we were able to determine the charge-carrier mobility μ according to
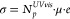
1where *e* is the elementary charge. P(g_3_2T-T), possessing the shortest side chains, exhibits the highest charge-carrier mobility μ = 18.7 ± 5.6 cm^2^ V^–1^ s^–1^, which is one order of magnitude larger than values obtained for the other two polymers (Table 1; the use of *N*_*p*_^*FTIR*^ yields a similar trend; cf. Table S1). The higher charge-carrier mobility of p(g_3_2T-T) compared to values obtained for the other two polymers is also consistent with a shift of the polaronic band in the infrared part of the UV–vis–IR absorption spectrum to lower energies. A shift of the IR polaronic absorption to lower energies has been shown to arise due to increased delocalization of polarons leading to a higher μ.^36^ We argue that the higher degree of π-stacking of doped p(g_3_2T-T) enhances the polaron delocalization.

**Figure 5:**
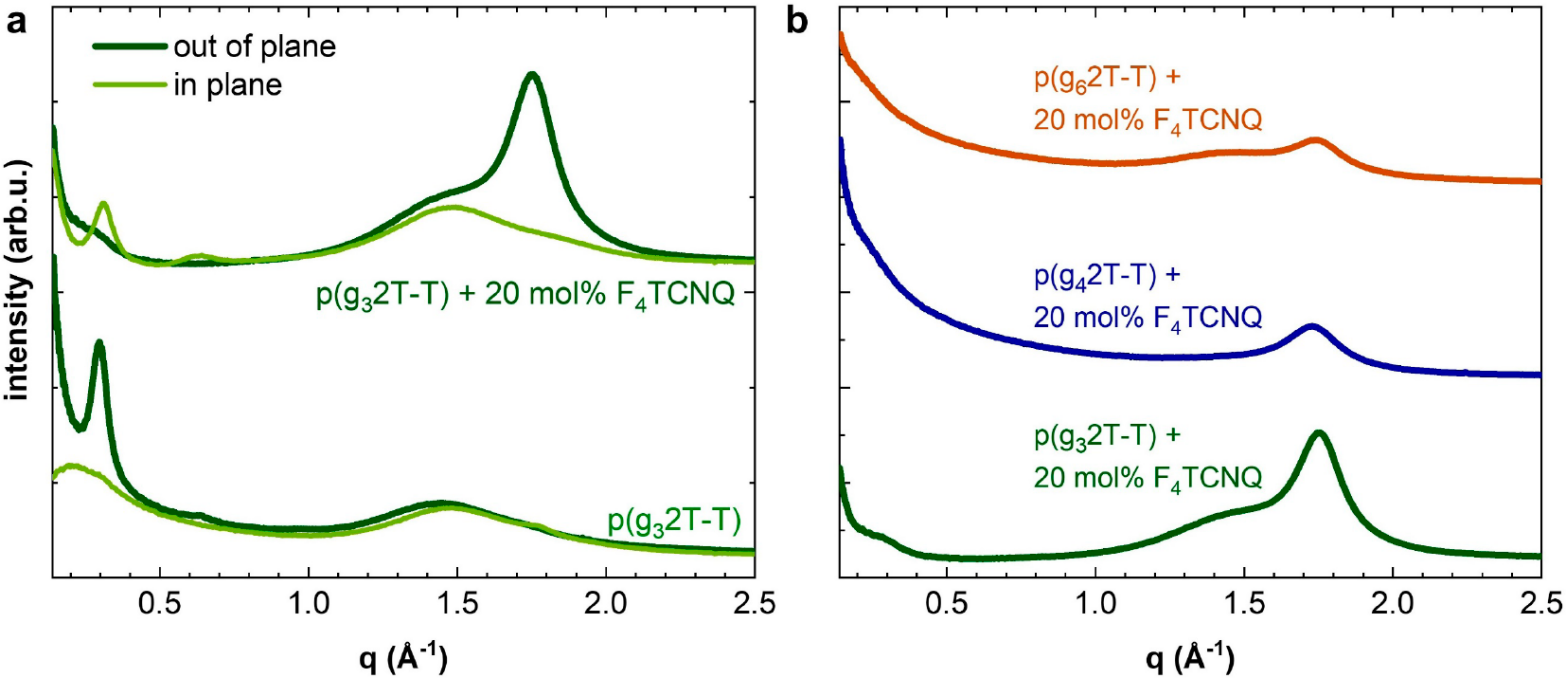
(a) In-plane (light green) and out-of-plane (dark green) GIWAXS diffractograms of neat p(g_3_2T-T) and coprocessed with 20 mol % of F_4_TCNQ. (b) Out-of-plane GIWAXS diffractograms of p(g_*x*_2T-T) polymers coprocessed with 20 mol % of F_4_TCNQ.

Grazing-incidence wide-angle X-ray scattering (GIWAXS) was carried out to determine the impact of the side-chain length on the degree of order of the polymers (see Figure S6 for GIWAXS 2D patterns). GIWAXS diffractograms of neat p(g_3_2T-T) films feature out-of-plane *h*00 diffraction peaks (*h* = 1–3) with a distinct peak at *q*_100_ ≈ 0.30 Å^–1^, typical for an edge-on texture of the polymer backbone. A weak in-plane *q*_010_ diffraction is observed, indicating limited π-stacking on top of a broad amorphous halo at *q* ≈ 1.5 Å^–1^ (Figure 5). The addition of 20 mol % F_4_TCNQ results in a change in texture from a predominately edge-on to face-on orientation, as evidenced by in-plane *h*00 diffraction peaks. Moreover, a distinct out-of-plane diffraction peak *q*_010_ ≈ 1.75 Å^–1^ emerges due to the π-stacking of the polymer backbone. GIWAXS diffractograms of the other two polymers with longer side chains, p(g_4_2T-T) and p(g_6_2T-T), exhibit no change in the texture when doped. They show a predominant face-on orientation with no *h*00 diffraction, in both their neat and oxidized states, indicating that longer side chains hinder lamellar stacking to a large extent. Upon doping with 20 mol % F_4_TCNQ, an out-of-plane diffraction peak *q*_010_ appears for both polymers, analogous to p(g_3_2T-T) oxidized with the same amount of dopant. The intensity of the out-of-plane *q*_010_ diffraction peak decreases with increasing side-chain length (GIWAXS of doped films in Figure 5b and WAXS of doped bulk samples in Figure S7), indicating a higher degree of order for the polymer with the shortest side chains, i.e., p(g_3_2T-T). Using the full-width at half-maximum (FWHM) Δθ_*FWHM*_ of the *q*_010_ diffraction peak, we estimated the π-stacking coherence length *L*_*c*_ for the three doped polymers using the Scherrer equation:
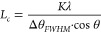
2where *K* = 0.9 is a shape factor, λ = 1 Å is the X-ray wavelength, and θ is the peak diffraction angle. We obtain a value of *L*_*c*_ ≈ 7.4 ± 0.4 nm for p(g_3_2T-T), larger than *L*_*c*_ ≈ 5.0 ± 0.6 and 5.1 ± 0.4 nm for p(g_4_2T-T) and p(g_6_2T-T), respectively. The higher coherence length of ordered domains in case of doped p(g_3_2T-T) is consistent with the higher deduced charge-carrier mobility (see Table 1).^37^

In a further set of experiments, we explored the thermoelectric properties of p(g_*x*_2T-T) polymers coprocessed with 20 mol % F_4_TCNQ. The Seebeck coefficient values for all three polymers are similar, around α = 14 to 16 μV K^–1^, indicating that the length of the side chains does not affect the thermovoltage (Table 1 and Figures 4, S5). These values, together with the electrical conductivity, result in a power factor of up to α^2^σ = 20.7 ± 3.8 μW m^–1^ K^–2^ for p(g_3_2T-T) and lower values of α^2^σ = 1.1 ± 0.1 μW m^–1^ K^–2^ for p(g_4_2T-T) and α^2^σ = 1.0 ± 0.1 μW m^–1^ K^–2^ for p(g_6_2T-T) (Figures 4 and S5).

We monitored the thermoelectric properties over time to study the stability of doped p(g_*x*_2T-T) polymers under ambient conditions (Figures 4 and S5). All three doped polymers are sensitive to air, as evidenced by a drop in electrical conductivity within the first 24 h of aging, thus leading to a decrease in the thermoelectric performance. After an initial drop in σ, the thermoelectric properties of all three polymers display a promising level of long-term stability. For instance, after aging for three months at ambient conditions, thin films of p(g_3_2T-T) coprocessed with F_4_TCNQ retain a high σ ≈ 200 S cm^–1^, α ≈ 16 μV K^–1^, and hence, α^2^σ ≈ 5 μW m^–1^K^–2^. The initial drop in electrical conductivity can be attributed to partial dedoping that occurs under ambient conditions. UV–vis spectra of samples aged for 3 months clearly show the reappearance of the absorption peak of the neat polymer, along with a reduction in intensity of the polaronic absorption bands (Figure S8). Additionally, the peak at 3.4 eV assigned to neat F_4_TCNQ decreases, which we explain with gradual sublimation of the dopant under ambient conditions.

Frequency-domain thermoreflectance was used to evaluate the out-of-plane thermal conductivity κ of p(g_3_2T-T) in its neat and oxidized state. We observed a reduction from κ = 0.39 ± 0.01 W m^–1^ K^–1^ for the neat polymer to κ = 0.23 ± 0.02 W m^–1^ K^–1^ when coprocessed with 20 mol % F_4_TCNQ. Despite the high electrical conductivity of doped p(g_3_2T-T), which could suggest a significant electronic contribution to the total κ depending on the specific Lorenz factor, an increase in thermal conductivity upon doping is not observed. In case of F_4_TCNQ-doped regioregular P3HT, oriented by solid-state pressing, κ was found to be lowest along the lamellar stacking direction but showed similar values along the backbone orientation and π-stacking direction.^38^ In contrast, upon doping, p(g_*x*_2T-T) films show a preferential face-on orientation, i.e., in-plane lamellar stacking, but nevertheless feature a decrease in the out-of-plane thermal conductivity. A decrease in the out-of-plane thermal conductivity has also been observed in case of poly(2,5-bis(3-tetradecylthiophen-2-yl)thieno[3,2-*b*]thiophene) (PBTTT) upon doping with F_4_TCNQ, which was attributed to a reduction in the lattice thermal conductivity caused by alloy scattering.^12^ Using the measured out-of-plane thermal conductivity value of κ = 0.23 ± 0.02 W m^–1^ K^–1^ and a power factor of α^2^σ = 20.7 ± 3.8 μW m^–1^ K^–2^, we estimate an upper limit for the figure of merit of *ZT* = 0.027 ± 0.005 at 300 K for as-cast p(g_3_2T-T) films coprocessed with 20 mol % F_4_TCNQ, which decreases to a value of *ZT* = 0.007 ± 0.002 at 300 K for samples aged for 3 months at ambient conditions. We note that even though aged films are partially dedoped, the polymer continues to be strongly oxidized, and therefore, we expect κ to remain low.

**Figure 6:**
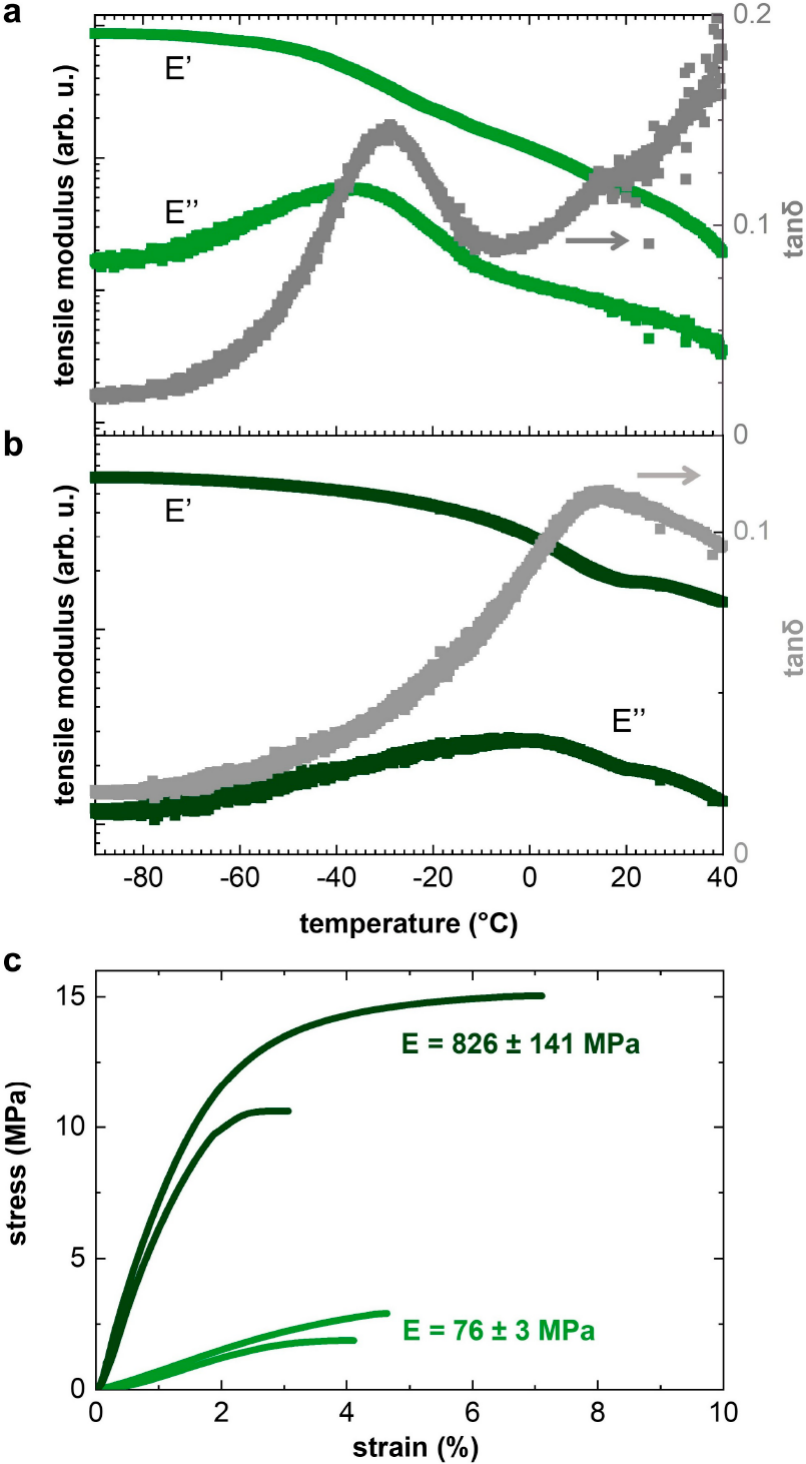
DMA thermograms of the storage and loss modulus, *E*′ and *E*″, and tan δ of (a) neat p(g_3_2T-T) and (b) p(g_3_2T-T) coprocessed with 20 mol % of F_4_TCNQ. (c) Stress–strain curves recorded by tensile deformation of free-standing films of neat p(g_3_2T-T) (light green) and p(g_3_2T-T) doped with 20 mol % F_4_TCNQ (dark green).

In a final set of experiments, we investigated the mechanical properties of p(g_3_2T-T) in both its neat and oxidized states. Dynamic mechanical analysis (DMA) of neat p(g_3_2T-T) supported by a glass fiber mesh (Figure 6a) indicates a softening of the material upon heating from −90 to 40 °C, i.e., the storage modulus *E*′ decreases and the loss modulus *E*″ shows a peak at −36 °C, which we assign to the glass transition temperature *T*_*g*_. This temperature corresponds to a transition from the glassy to the rubbery state of the material due to the onset of main-chain relaxation, possibly accompanied by the onset of relaxation of the triethylene glycol side chains.^20,39,40^ DMA of p(g_4_2T-T) and p(g_6_2T-T) (Figure S9) indicates similar values of *T*_*g*_ = −41 and −43 °C, respectively. Similar to poly(3-alkylthiophene)s (P3ATs) with long alkyl side chains (e.g., decyl, dodecyl), an increase in the side-chain length from triethylene glycol to tetra- or hexaethylene glycol only slightly reduces the *T*_*g*_.^41^

Doping of p(g_3_2T-T) with 20 mol % F_4_TCNQ considerably increased the *T*_*g*_ from −36 to 1 °C (Figure 6b). The increase in *T*_*g*_ can be explained with the doping induced π-stacking of the polymer (see GIWAXS diffractograms in Figure 4), as also argued previously for doped p(g_4_2T-T), and with the quinoidal structure of the oxidized backbone.^20,23^ We used tensile deformation of free-standing samples at room temperature to analyze the mechanical properties of neat and doped p(g_3_2T-T) (Figure 6c). The neat polymer displays a Young’s modulus of *E* = 76 ± 3 MPa and strain at break of ε_*b*_ = 4.3 ± 0.2%, indicating a relatively soft but brittle material. Instead, p(g_4_2T-T) has been reported to have a lower *E* of 1 to 8 MPa.^20,40^ Evidently, shortening of the oligoether side chains enhances the stiffness of the polymer, as also observed for P3ATs.^23^ When the polymer is coprocessed with 20 mol % F_4_TCNQ, the material becomes significantly stiffer, with a Young’s modulus *E* = 826 ± 141 MPa, but retains a similar ε_*b*_ = 5 ± 2%. This result aligns with recent studies that highlight molecular doping as an effective tool for tuning not only the electrical but also the mechanical properties of conjugated polymers.^20,34^

## Conclusions

The side-chain length of p(g_*x*_2T-T) type polythiophenes considerably influences the ability of the polymer to order. The shortest investigated side chains, triethylene glycol, yielded a more ordered polymer, in particular when coprocessed with the p-dopant F_4_TCNQ. The high degree of π-stacking of oxidized p(g_3_2T-T) resulted in a stiff material with promising thermoelectric properties. A high electrical conductivity of up to σ = 830 ± 15 S cm^–1^ was observed for as-cast films, which together with a Seebeck coefficient of α = 15.8 ± 2.0 μV K^–1^ resulted in a promising thermoelectric power factor of α^2^σ = 20.7 ± 3.8 μW m^–1^ K^–2^. Aging at ambient conditions resulted in an initial drop in σ to a value of about 200 S cm^–1^, which subsequently remained stable for at least 3 months. It can be concluded that identifying the optimal side-chain length of polar polythiophenes is an effective strategy for improving their thermoelectric and mechanical properties.

## Materials and Methods

### Materials

P(g_3_2T-T) (*M*_*n*_ = 32 kg mol^–1^ and polydispersity index PDI = 2.5), p(g_4_2T-T) (*M*_*n*_ = 24 kg mol^–1^ and PDI = 3.3), and p(g_6_2T-T) (*M*_*n*_ = 28 kg mol^–1^ and PDI = 5.1) were synthesized according to reported procedures.^14,16,20^ F_4_TCNQ was purchased from Tokyo Chemical Industry (TCI) and used as received. Chloroform (CHCl_3_, Fisher Scientific, purity > 99.8%), chlorobenzene (CB, Sigma-Aldrich, purity > 99.5%), acetonitrile (AcN, VWR Chemicals, HPLC-super gradient), and dimethylformamide (DMF, Sigma, HPLC-grade, purity ≥ 99.9%) were used as received.

### Size Exclusion Chromatography (SEC)

Chromatograms were recorded using an Agilent 1260 Infinity GPC running at an oven temperature of 70 °C, employing two columns and a precolumn containing a Polargel M 300 × 7.5 mm with mixed pores and a pore size of 8 μm. Polymer samples were dissolved in DMF at a concentration of about 1 g L^–1^. The eluent used was DMF with 0.1 wt % LiBr (Sigma, Reagentplus, purity ≥99.9%). Relative calibration was carried out with poly(methyl methacrylate) standards.

### Sample Preparation

Thin films for spectroscopy and electrical characterization were prepared by adding solutions of F_4_TCNQ in AcN (2 g L^–1^) to solutions of p(g_3_2T-T), p(g_4_2T-T), or p(g_6_2T-T) in CHCl_3_ (4 g L^–1^) to obtain different polymer:dopant ratios, together with further AcN or CHCl_3_ to ensure a final solvent ratio of 2:1 CHCl_3_:AcN. The molar percentage of F_4_TCNQ was calculated with respect to the number of thiophene rings of the conjugated polymers. The polymer:dopant solution was then bar-coated or spin-coated at 1000–1500 rpm for 60 s onto precleaned microscopy glass slides for UV–vis spectroscopy, CaF_2_ substrates for FTIR spectroscopy, PET films for Seebeck measurements, or silicon substrates (cleaned with acetone and isopropanol and treated by plasma/ozone for 5 min) for GIWAXS and thermal conductivity measurements. Free-standing samples with a thickness of 20 to 70 μm for mechanical testing were made by drop casting solutions of p(g_3_2T-T) in 2:8 CB:CHCl_3_ (10 g L^–1^), p(g_4_2T-T) in CHCl_3_ (10 g L^–1^), or a mixture of polymer and dopant solution (F_4_TCNQ dissolved at 2 g L^–1^ in AcN) at 35 °C onto glass slides followed by removal from the substrate with a sharp blade. Neat p(g_3_2T-T) and p(g_4_2T-T) samples were frozen in liquid nitrogen prior to removal of the polymer film from the substrate. Glass fiber supported samples were made through coating glass mesh strands cut at 45° with p(g_3_2T-T) (2:8 CB:CHCl_3_, 10 g L^–1^), p(g_4_2T-T) (CB, 10 g L^–1^), or p(g_6_2T-T) (CB, 10 g L^–1^), followed by drying at 30 °C under vacuum for 24–48 h. Bulk samples for WAXS measurements were drop cast at 35 °C onto glass slides. These were cast using solutions of p(g_*x*_2T-T) polymer dissolved at 20 g L^–1^ in CHCl_3_ for neat samples and a mixture of polymer (15 g L^–1^ in CHCl_3_) and F_4_TCNQ (2 g L^–1^ in AcN) solutions for doped ones.

### UV–vis Absorption Spectroscopy

A PerkinElmer Lambda 1050 spectrophotometer was used to record UV–vis–NIR spectra on thin films with a thickness of 25 to 100 nm.

### Fourier Transform Infrared Spectroscopy (FTIR)

A PerkinElmer FT-IR Spectrometer “Frontier” was used to perform infrared absorption measurements on thin F_4_TCNQ-doped p(g_*x*_2T-T) films coated on CaF_2_.

### Grazing-Incidence Wide-Angle X-ray Scattering (GIWAXS)

Films were prepared by spin-coating onto cleaned and plasma treated silicon wafers. GIWAXS diffractograms were recorded at the beamline NCD – SWEET of the Alba synchrotron light source facility using an X-ray wavelength of 1 Å and a sample–detector distance of 201.17 cm.

### Wide-Angle X-ray Scattering (WAXS)

Wide-angle X-ray scattering (WAXS) was carried out in transmission mode on bulk samples with a Mat:Nordic instrument from SAXSLAB equipped with a Rigaku 003+ high-brilliance microfocus Cu Kα radiation source (wavelength = 1.5406 Å) and a Pilatus 300 K detector placed at a distance of 124.6 mm from the sample.

### Electrical Characterization

The electrical resistance was measured with a four-point probe setup from Jandel Engineering (cylindrical probe head, RM3000) using collinear tungsten carbide electrodes at regular spacing of 1 mm. The electrical conductivity was calculated by taking into consideration the sample thicknesses and a geometrical factor of 4.53. The thickness of the thin films was measured by a Digital Instrument Dimension 3000 Large Sample AFM with a type G Scanner using a standard silicon tip in tapping mode. The Seebeck coefficient at 300 K was measured with an SB1000 instrument (MMR Technologies) equipped with a K2000 temperature controller (MMR Technologies) using a thermal load of 1–2 K and a Constantan wire as the internal reference. F_4_TCNQ-doped p(g_*x*_2T-T) solutions were spin coated on PET foil, cut into pieces of around 1 × 5 mm, and mounted on the SB1000 sample stage with carbon paint (DAG-T-502, Ted Pella).

### Dynamic Mechanical Analysis (DMA)

A Q800 dynamic mechanical analyzer from TA Instruments was used for mechanical characterization. Dynamic mechanical analysis (DMA) was performed at a frequency of 1 Hz while ramping the temperature from −90 to 140 °C (glass fiber mesh samples) and −100 to 60 °C (free-standing samples) at a rate of 3 °C min^–1^. A dynamic strain with a maximum value of 0.01–0.04% and a preload force of 0.002–0.004 N were used for samples supported by the glass fiber mesh, which were clamped with the glass fiber strands at 45° to the direction of deformation. A dynamic strain with a maximum value of 0.05% and a preload force of 0.005 to 0.01 N were used for free-standing samples. Tensile testing was performed at room temperature in controlled force mode with a force rate of 0.005 N min^–1^ using a preload force of 0.01 N and gauge length of 3.7–4.2 mm.

### Frequency-Domain Thermoreflectance (FDTR)

The thermal conductivity was measured by the frequency-domain thermoreflectance (FDTR) method.^42^ The setup consisted of two lasers to heat (pump) and probe (probe) the local temperature (spot size of ∼10 μm in diameter) at the sample surface. A 40 nm thick gold transducer was thermally evaporated onto the surface of the samples to limit the optical penetration and improve the thermal sensitivity of the method. The wavelengths of the pump and probe lasers were set to 405 and 532 nm, respectively. The output power of the pump laser was modulated to a harmonic waveform in the frequency range between 30 kHz and 40 MHz. The pump laser generates thermally induced harmonic oscillations of the reflectivity of the sample, thus leading to a modulation of the reflected power of the continuous wave probe laser. The phase lag between the pump and probe harmonic waves was measured by using a lock-in amplifier and modeled by numerically solving the parabolic heat equation. Thus, the cross-plane thermal conductivity was obtained by fitting the phase lag vs the applied frequency.

### Heat Capacity Measurements

The specific heat capacity of neat p(g_4_2T-T) was measured with a DSC2 instrument from Metter Toledo using a temperature-modulated DSC (TMDSC) method and a sapphire reference. Measurements were performed in the temperature range from 10 to 50 °C with a heating rate of 1 °C min^–1^. The DSC was calibrated in the same temperature region before each run, using a sapphire sample.

## Supporting Information Available

UV–vis absorbance and FTIR spectra, thermoelectric properties, charge-carrier density, GIWAXS patterns, WAXS diffractograms, DMA thermograms, and GPC traces (PDF)
