# Supplementary material for: Impact of Oligoether Side-Chain Length on the Thermoelectric Properties of a Polar Polythiophene
Source: ACS Appl Electron Mater. 2023 Sep 15;6(5):2909–16. doi: 10.1021/acsaelm.3c00936 (PMC11137803; doi:10.1021/acsaelm.3c00936)
Supplement: Supplementary file 1 — el3c00936_si_001.pdf [file el3c00936_si_001.pdf]

Supporting Information

Impact of oligoether side-chain length on the thermoelectric properties of a polar polythiophene

Mariavittoria Craighero[1], Jiali Guo[2], Sepideh Zokaei[1], Sophie Griggs[3], Junfu Tian[3], Jesika Asatryan[4], Joost Kimpel[1], Renee Kroon,[5] Kai Xu[2], Juan Sebastian Reparaz[2], Jaime Martín [4,6], Iain McCulloch[3], Mariano Campoy-Quiles[2], Christian Müller[1]*

[1]Department of Chemistry and Chemical Engineering, Chalmers University of Technology, Goteborg 41296, Sweden

*e-mail: christian.muller@chalmers.se

[2]Materials Science Institute of Barcelona, ICMAB-CSIC, Campus UAB, 08193 Bellaterra, Spain

[3]Department of Chemistry, University of Oxford, Chemistry Research Laboratory, 12 Mansfield Road, Oxford OX1 3TA, United Kingdom

[4] Universidade da Coruña, Campus Industrial de Ferrol, CITENI, Esteiro, 15403, Ferrol, Spain

[5]Laboratory of Organic Electronics, Linköping University, 60174, Norrköping, Sweden

[6] POLYMAT, Paseo Manuel de Lardizabal 3, 20018, Donostia-San Sebastián, Spain

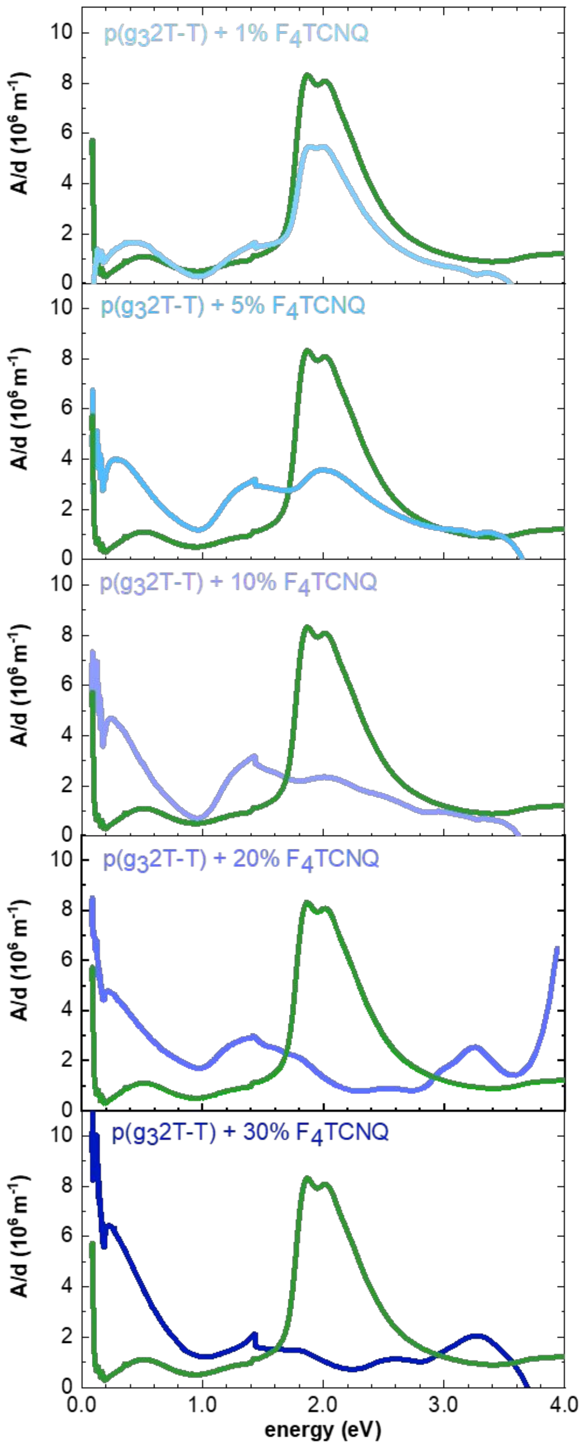

Figure S1. UV-vis absorbance spectra, with the absorbance *A* normalized by the film thickness *d*, of p($g_3$2T-T) before (green) and after co-processing with 1 mol%, 5 mol%, 10 mol%, 20 mol% and 30 mol% F_4TCNQ (blue).

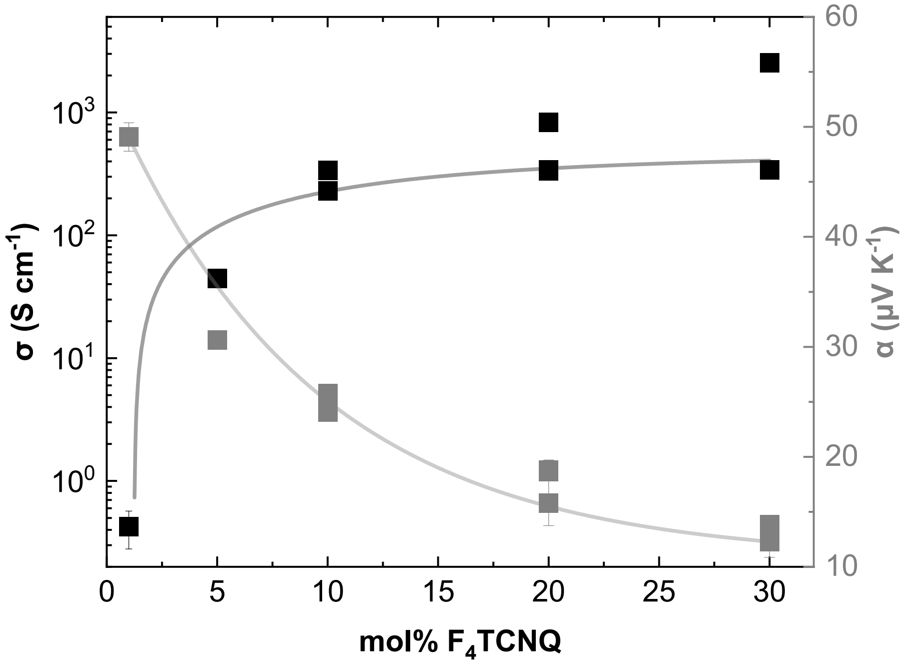

Figure S2. Thermoelectric properties of p($g_3$2T-T) co-processed with 1, 5, 10, 20 and 30 mol% F_4TCNQ.

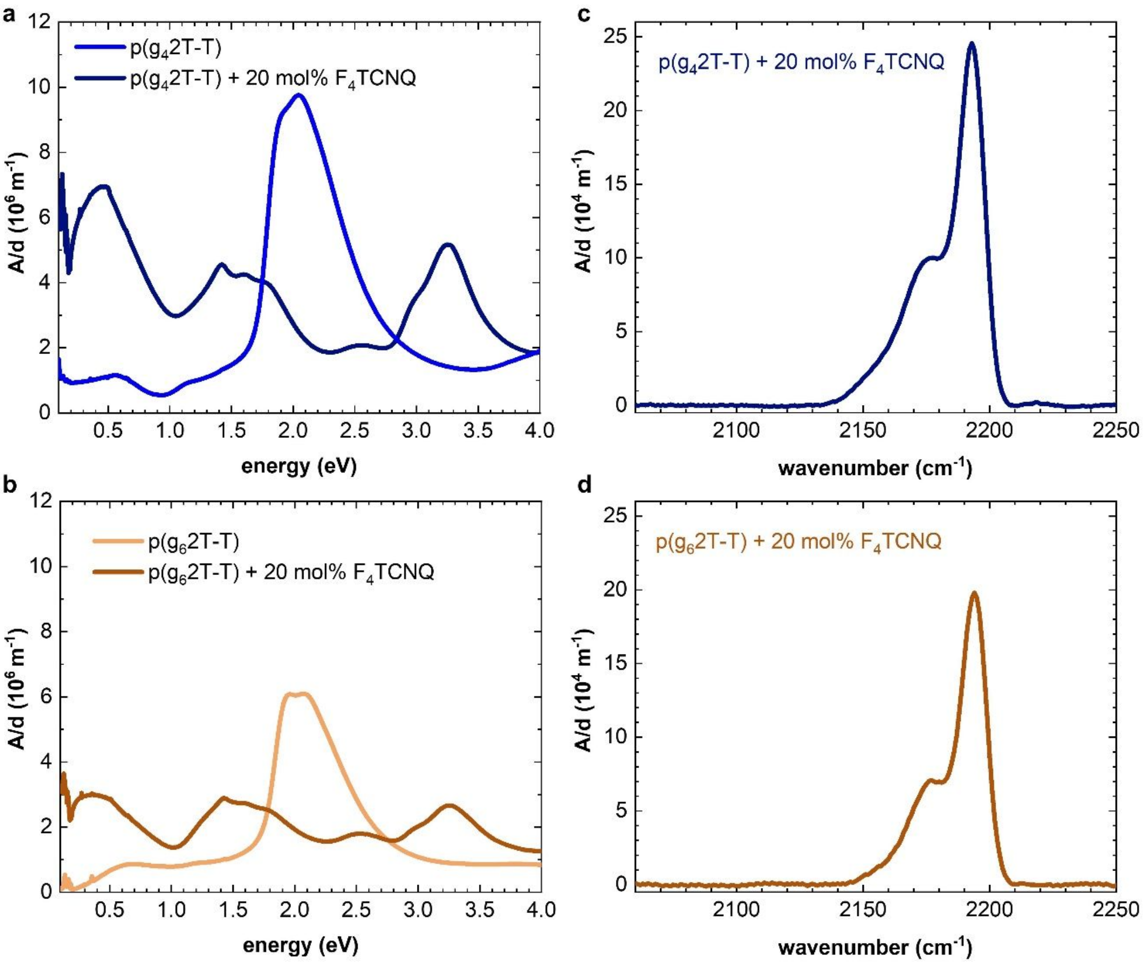

Figure S3. UV-vis (a,b) and transmission FTIR (c,d) absorbance spectra, with the absorbance A normalized by the film thickness d, of p(g_x2T-T) before (light color) and after co-processing with 20 mol% F_4TCNQ (dark color).

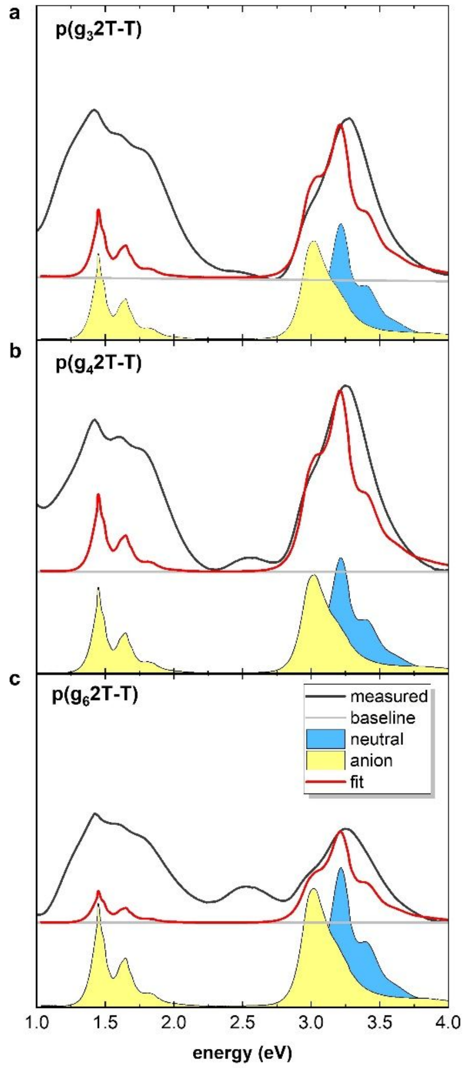

Figure S4. UV-vis spectra of p(g$_3$2T-T) (a), p(g$_4$2T-T) (b) and p(g$_6$2T-T) (c) films co-processed with 20 mol% F_4TCNQ (dark grey), of neutral F_4TCNQ and its anion in acetonitrile from Kiefer et al., Nature Mater. 2019, 18, 149-155 (blue and yellow filled curves) and fit of the measured spectra in the UV region composed of the spectra of neutral F_4TCNQ and its anion as well as a vertical offset (red). The experimental spectra were fitted with a superposition of a horizontal baseline (grey) and the spectra of neutral F_4TCNQ and its anion (blue and yellow). The ratio of ionized and neutral dopant molecules f was calculated by comparing the contributions of their respective UV-vis spectra to the fit.

Calculation of number of charge carriers (polarons) per unit volume

The number of charge carriers (polarons) N_p^{UVvis} per unit volume was estimated according to:

$$N_p^{UVvis} = \eta_{ion} \cdot x_d \left(\frac{M_{g_x 2T-T}}{3 \cdot \rho_{p(g_x 2T-T)}} (100 - x_d) + \frac{M_{F_4TCNQ}}{\rho_{F_4TCNQ}} x_d \right)^{-1} \quad \text{(S1)}$$

where x_d is the dopant molar concentration (i.e. 20 mol% F_4TCNQ), $\rho_{p(g_x 2T-T)}$ = 1 g cm^{-3} and ρ_{F_4TCNQ} = 1.6 g cm^{-3} are the assumed density of p(g_x2T-T) and F_4TCNQ, and $M_{g_x 2T-T}$ = 570, 659 or 835 g mol^{-1} and M_{F_4TCNQ} = 276 g mol^{-1} are the molecular weight of the g_x2T-T repeat unit and F_4TCNQ.

Table S1. Electrical properties of thin films co-processed with 20 mol% F_4TCNQ per thiophene ring: polymer, number of polarons N_p^{FTIR} per unit volume (estimated error of 30% from the thickness measurement and the applied fitting procedure) and ionization efficiency η_{ion}^{FTIR} from analysis of FTIR spectra, electrical conductivity σ (error represents the standard deviation of five measurements on the same sample), charge mobility μ.

polymer	N_p^{FTIR} (10^{26} m^{-3})	η_{ion}^{FTIR} (%)	σ (S cm^{-1})	μ (cm^2 V^{-1} s^{-1})
p($g_3$2T-T)	2.2 ± 0.7	31 ± 9	830 ± 15	23.9 ± 7.2
p($g_4$2T-T)	4.2 ± 1.3	60 ± 18	56 ± 3	0.8 ± 0.3
p($g_6$2T-T)	3.3 ± 1.0	47 ± 14	51 ± 4	1.0 ± 0.3

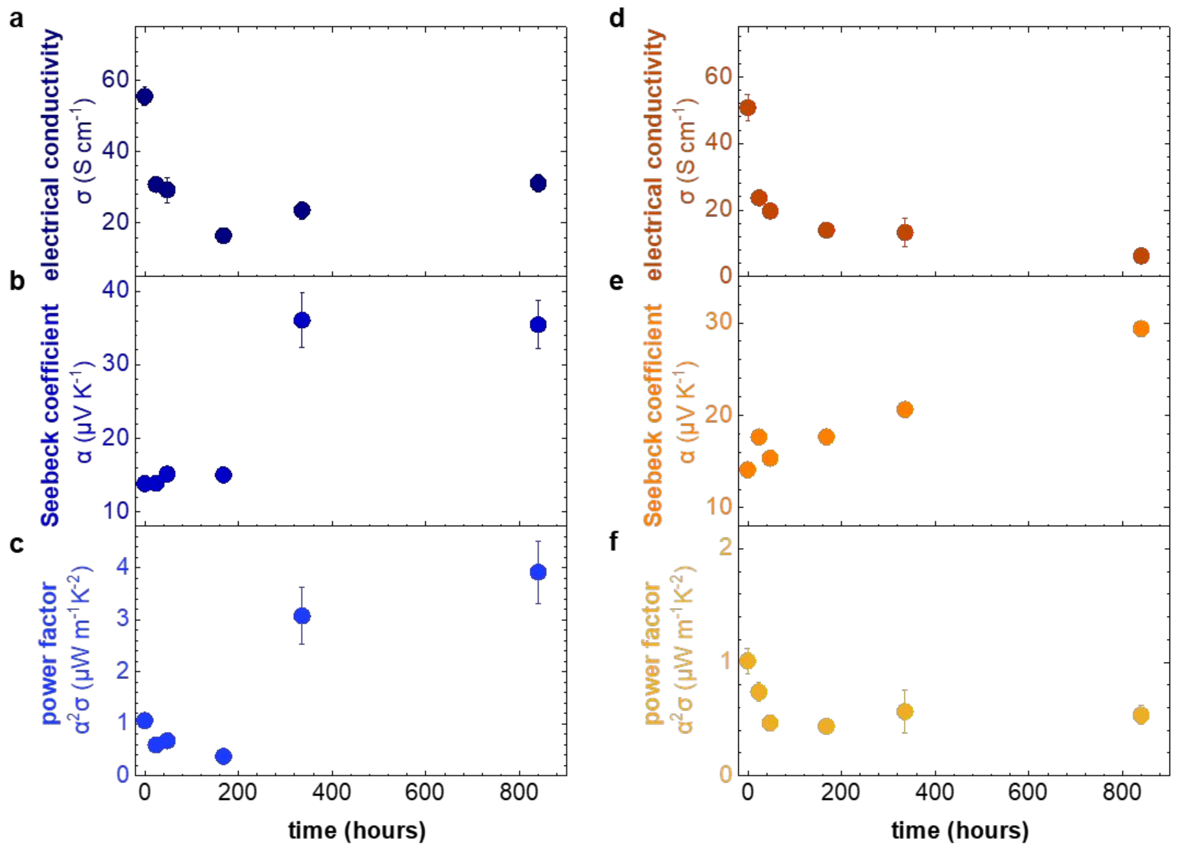

Figure S5. Electrical conductivity (a,d), Seebeck coefficient (b,e) and power factor (c,f) vs aging time of p($g_4$2T-T) and p($g_6$2T-T) co-processed with 20 mol% of F_4TCNQ as-cast and aged at ambient conditions.

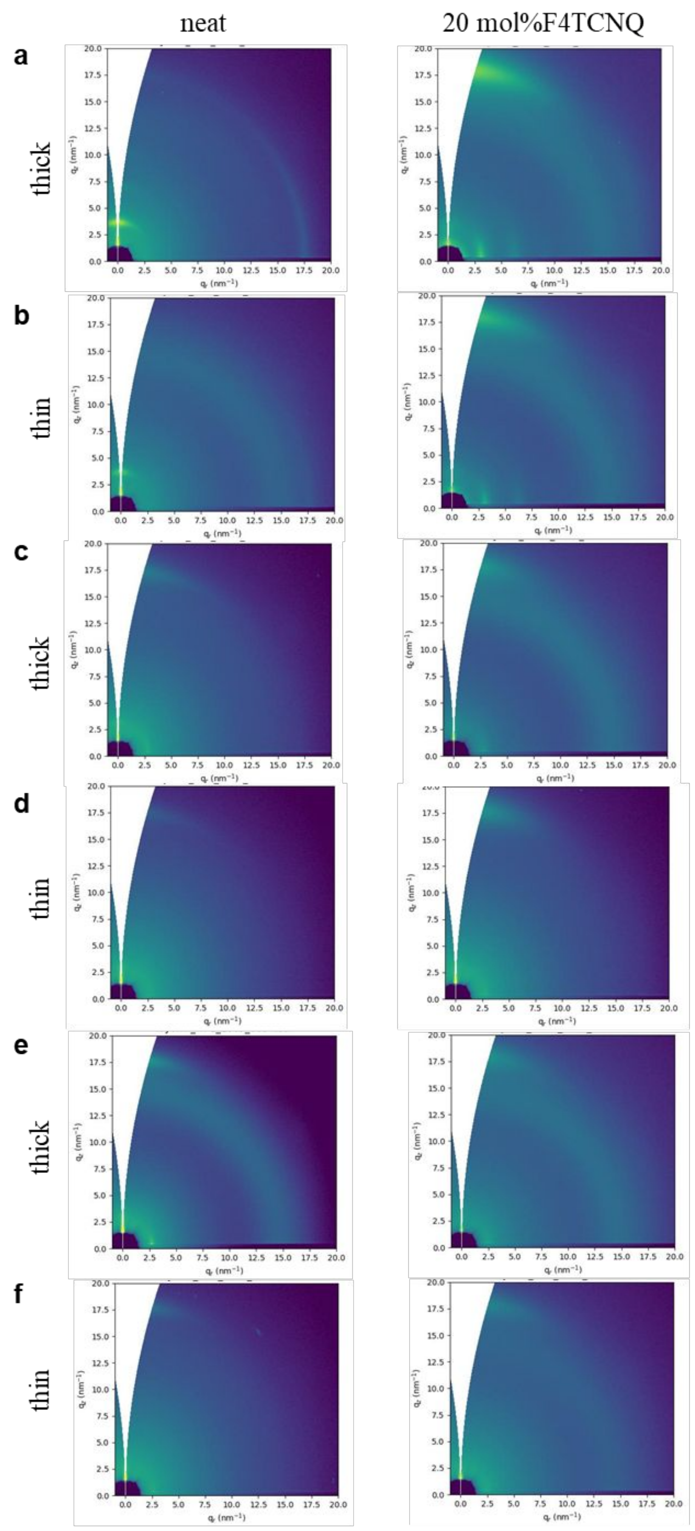

Figure S6. GIWAXS patterns of p($g_3$2T-T) (a,b), p($g_4$2T-T) (c,d) and p($g_6$2T-T) (e,f) neat and co-processed with 20 mol% F_4TCNQ.

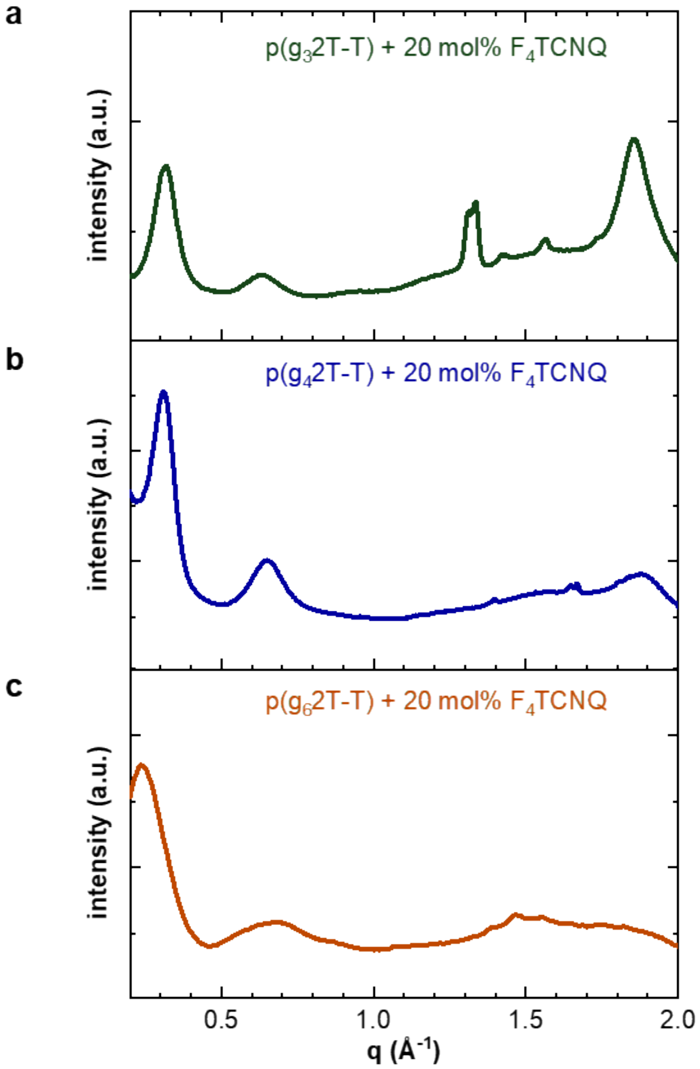

Figure S7. Transmission WAXS diffractograms of (a) p(g$_3$2T-T), (b) p(g$_4$2T-T) and (c) p(g$_6$2T-T) (c) co-processed with 20 mol% F$_4$TCNQ. The additional peaks between 1.3 and 1.6 Å^{-1} likely arise because of the presence of excess F$_4$TCNQ.

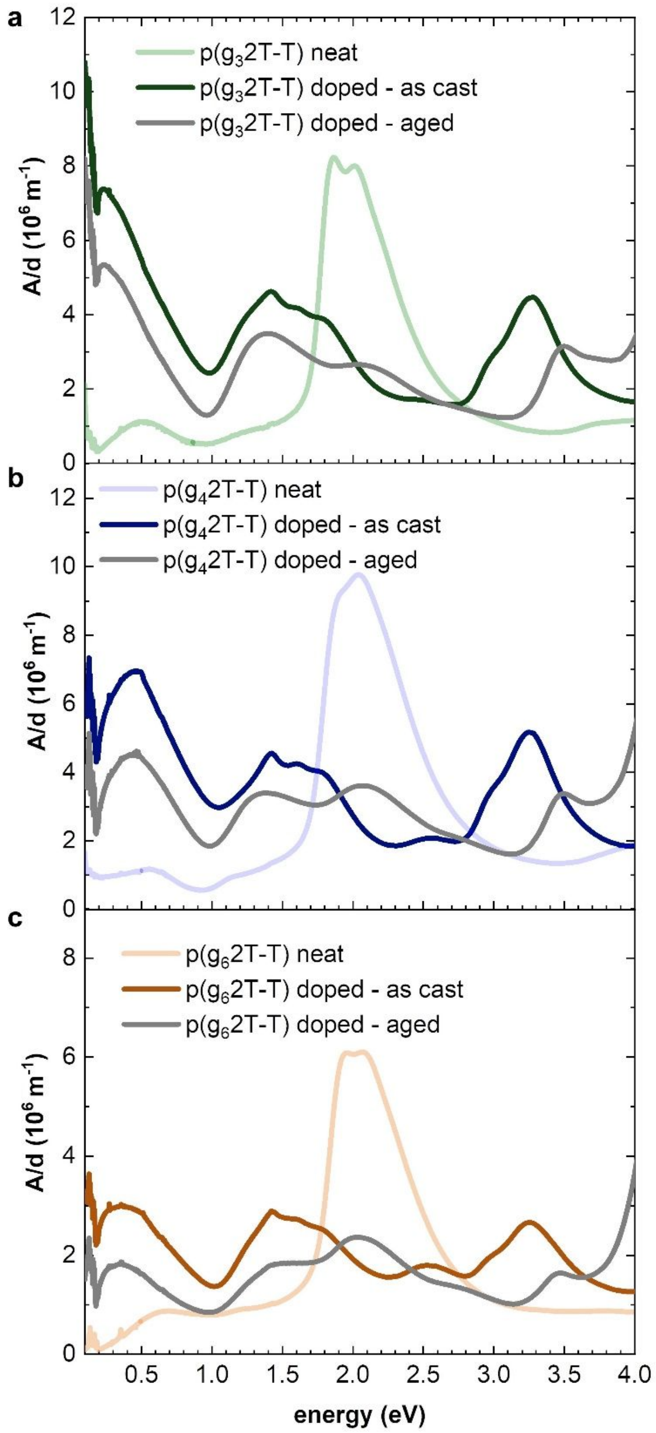

Figure S8. UV-vis and transmission FTIR absorbance spectra, with the absorbance *A* normalized by the film thickness *d*, of p($g_3$2T-T) (a), p($g_4$2T-T) (b) and p($g_6$2T-T) (c) neat and doped with 20 mol% F_4TCNQ samples as-cast and aged at ambient conditions for 3 months.

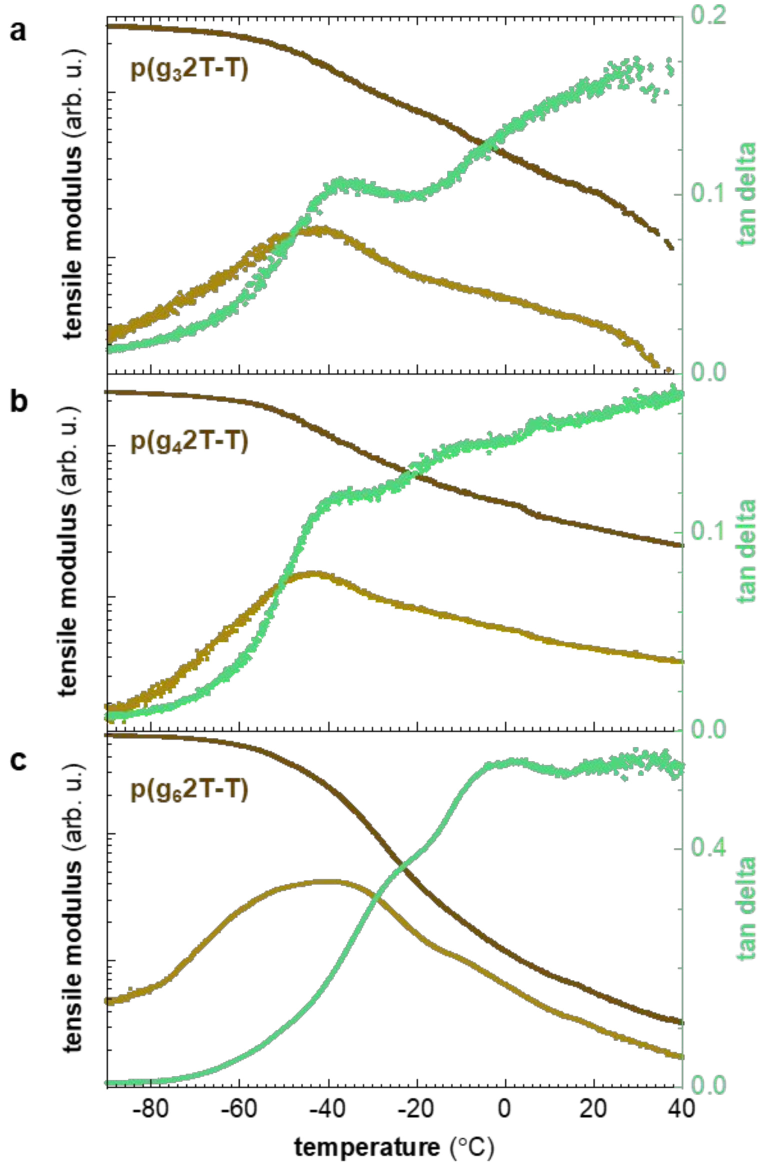

Figure S9. Tensile storage (dark brown) and loss (light brown) modulus, *E'* and *E''*, and $tan\delta = E''/E'$ (green) of neat p($g_3$2T-T) (a), p($g_4$2T-T) (b) and p($g_6$2T-T) (c) recorded as a function of temperature on samples supported by a glass fiber mesh.

a

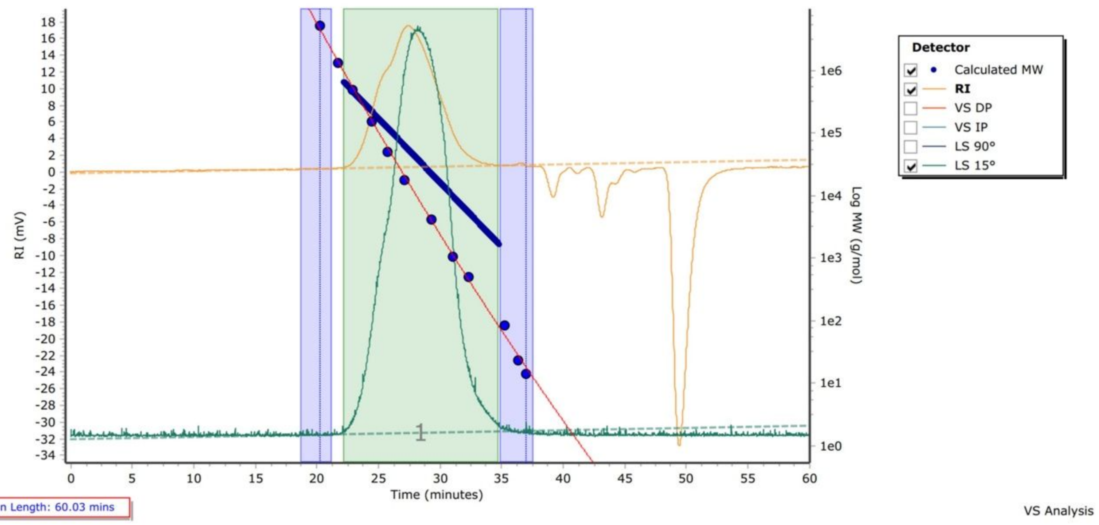

b

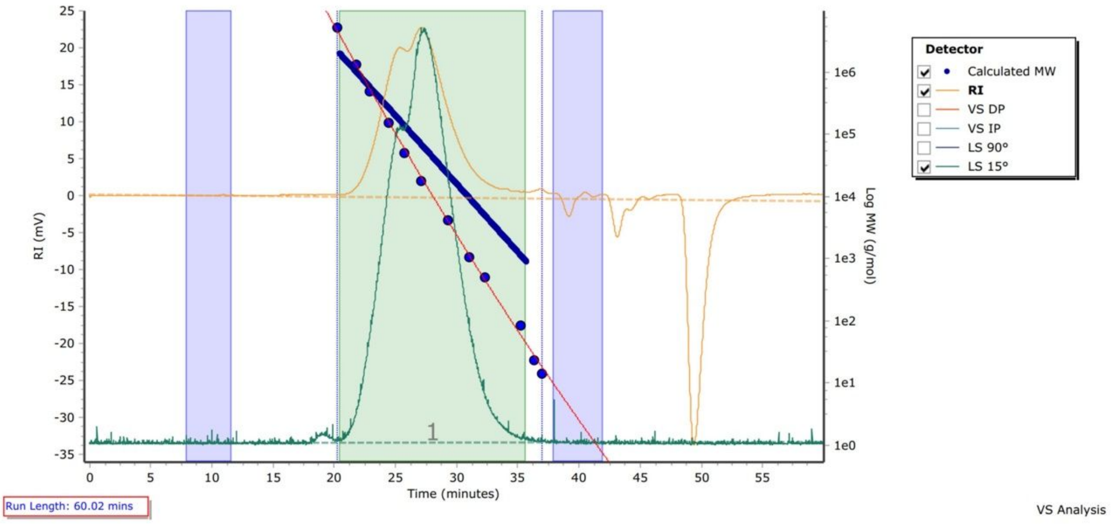

Figure S10. GPC traces of p($g_3$2T-T) (a) and p($g_6$2T-T) (b) at 343 K in DMF with 0.1 wt% LiBr at a polymer concentration of 1 mg mL^{-1}. Detectors showcased in traces are refractive index (orange) and light scattering (green).
